# CT-based muscle mass cutoff values for Caucasians according to the European Working Group on Sarcopenia recommendations

**DOI:** 10.1007/s00330-026-12396-9

**Published:** 2026-03-12

**Authors:** Natalie Wotschel, Tobias Schröter, Brenda Kociu, Hendrik Liebscher, Verena Plodeck, Heiner Nebelung, Ralf-Thorsten Hoffmann, Jürgen Weitz, Johanna Kirchberg, Felix Merboth

**Affiliations:** 1 Institute and Polyclinic for Diagnostic and Interventional Radiology, Faculty of Medicine and University Hospital Carl Gustav Carus, Technische Universität Dresden, Dresden, Germany; 2 Department of Visceral, Thoracic and Vascular Surgery, University Hospital and Faculty of Medicine Carl Gustav Carus, Technische Universität Dresden, Dresden, Germany; 3 National Center for Tumor Diseases (NCT/UCC), Dresden, Germany; 4 German Cancer Research Center (DKFZ), Heidelberg, Germany; 5 University Hospital and Faculty of Medicine Carl Gustav Carus Technische Universität Dresden, Dresden, Germany; 6 Helmholtz-Zentrum Dresden-Rossendorf (HZDR), Dresden, Germany

**Keywords:** Sarcopenia, Cutoff value, CT scan, Caucasian

## Abstract

**Objective:**

This study aimed to establish sex-specific cutoff values for sarcopenia based on the muscle parameters skeletal muscle index (SMI) and psoas muscle thickness per height (PMTH) using CT scans in a presumably healthy Caucasian study cohort.

**Materials and methods:**

In this retrospective, cross-sectional study, CT scans of 350 Caucasian patients (mean age, 32 years ± 8; mean BMI, 23 (women) and 24 kg/m^2^ (men)) with polytrauma, but otherwise young and presumably healthy, were evaluated. Sex-specific PMTH at the level of the umbilicus (mm/m) and the SMI (cm^2^/m^2^) at the level of the L3 vertebral body were calculated from cross-sectional images. Two standard deviations below the mean were considered the cutoff values for sarcopenia according to the recommendations of the European Working Group on Sarcopenia in Older People (EWGSOP). To exclude random outliers, only the 5th–95th percentiles were analyzed.

**Results:**

The sex-specific cutoff values for PMTH were 15.7 mm/m for women and 20.2 mm/m for men, while those for SMI were 33.2 and 44.0 cm^2^/m^2^, respectively. The intra-rater and inter-rater variabilities were consistently low, indicating the high reliability of the measurement method.

**Conclusions:**

This study established sex-specific thresholds for sarcopenia based on two different muscle parameters in a presumably healthy Caucasian study cohort according to EWGSOP recommendations.

**Key Points:**

***Question*** *Establishment of sarcopenia cutoff values according to the recommendations of the European Working Group on Sarcopenia in Older People (EWGSOP).*

***Findings*** *Cutoff values for psoas muscle thickness per height (mm/m) were 15.7 (women) and 20.2 (men); for skeletal muscle index (cm*^*2*^*/m*^*2*^*), 33.2 and 44.0, respectively.*

***Clinical relevance*** *These cutoff values for two different parameters that can be determined by CT allow patients with low muscle mass to be objectively identified, thereby revealing those at high risk of manifest sarcopenia.*

**Graphical Abstract:**

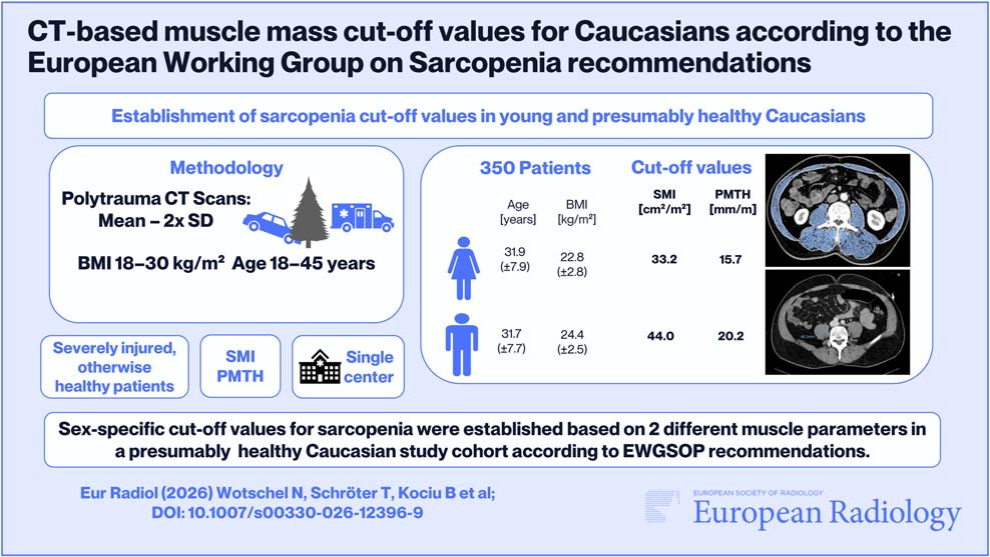

## Introduction

Sarcopenia is defined as a general reduction in skeletal muscle mass (SMM) and strength that increases with age or occurs secondary to other diseases [1–3]. Many chronic and malignant diseases commonly lead to the development of sarcopenia due to catabolic metabolic states [4, 5]. For example, the prevalence of sarcopenia at the initial diagnosis of a malignant disease is approximately 40–50% [4, 6]. In this context, sarcopenia is a known risk factor for adverse disease and treatment outcomes [7–10].

To diagnose sarcopenia, it is necessary to determine the muscle mass. Bioelectrical impedance analysis and dual-energy X-ray absorptiometry are used for this purpose. However, because both methods are inaccurate and error-prone, international working groups such as the European Working Group on Sarcopenia in Older People (EWGSOP) and Asian Working Group for Sarcopenia recommend CT as the gold standard at least for individuals at high risk of adverse outcomes [1, 2, 11]. CT scans are standard examinations, especially in oncological patients, so they are available for the analysis of muscle mass without additional expense or patient risk [12]. Common parameters used to quantify SMM on CT scans are the skeletal muscle index (SMI, cm^2^/m^2^) [12–15] and psoas muscle thickness per height (PMTH, mm/m) [16]. A strong association between the SMI and the SMM of the whole body has been previously reported [12, 16–19].

Numerous cutoff values for the SMI [1, 2, 9, 12–15, 20, 21] and PMTH [16, 19] have been established, with both cutoff values and underlying cohorts varying substantially between different studies. For example, for the SMI, cutoff values between 27.8 and 38.5 cm^2^/m^2^ for women and between 38.67 and 52.4 cm^2^/m^2^ for men have been published [22, 23]. In addition, the methods used to set the cutoff values also vary considerably. Thus, most research groups have used optimal stratification in terms of adverse events, postoperative complications, and mortality [11, 24, 25]. Therefore, the interpretation of these muscle parameters is limited.

Hence, the EWGSOP has recommended cutoff values for skeletal muscle indices that are two standard deviations (SD) below the mean of a healthy, young adult population [20], similar to bone densitometry and osteoporosis diagnosis. Further knowledge of these cutoff values for muscle mass could help identify and adequately treat patients with or at risk of sarcopenia. Therefore, this study aimed to establish sex-specific cutoff values for muscle mass based on the skeletal muscle parameters SMI and PMTH measured by CT in a presumably healthy, young, Caucasian population using mean values and SD.

## Materials and methods

### Patient characteristics

This retrospective, cross-sectional study was conducted at a single tertiary center. The study protocol was reviewed by a local ethics committee (BO-EK109032022) and was conducted in accordance with the Declaration of Helsinki and its subsequent amendments. A waiver was granted for informed consent.

All data were collected as part of routine guideline-guided care for patients with severe injuries or polytrauma. Polytrauma was defined as “a simultaneous injury to several body regions or organ systems, of which at least one or more injuries in combination are life-threatening” [26]. The patients underwent a prompt whole-body CT scan with a trauma-specific protocol as part of the diagnosis of severely injured patients according to the guidelines of the German Society for Trauma Surgery [27]. All consecutive CT scans obtained between 2018 and 2022 at our institute were included in the analysis.

Medical examinations of patients with polytrauma included a detailed history, physical examination, blood and urine tests, and imaging procedures, if necessary. The following study cohort was considered representative of the healthy population. In addition to the presence of (1) a polytrauma CT suitable for measurements, other inclusion criteria were as follows: (2) BMI between 18 and 30 kg/m^2^, (3) age of 18 to 45 years, and (4) Caucasian background, collected from the patient’s medical record. Exclusion criteria were as follows: (1) low-quality or incomplete CT scan, (2) abdominal trauma at the level of measurements, (3) pre-existing diseases according to the patient’s medical record, (4) alcohol or drug addiction, and (5) incomplete medical history regarding the inclusion and exclusion criteria.

### CT scans

CT scans were performed using a 128-line CT scanner (Somatom Definition Edge; Siemens Healthineers AG) according to the German polytrauma guidelines [27] and to the local CT protocol for polytrauma in the supine position. Contrast-enhanced images with 3-mm slice thickness were selected for measurements. The following CT parameters were used: rotation time, 0.5 s; pitch, 0.6; collimation, 128 × 0.6 mm; effective current, 210 mAs; voltage, 120 kV; reconstruction using B30f kernel; and matrix size, 512 × 512. The contrast agent was administered as a split bolus: 60 mL Ultravist 370 (Bayer Vital GmbH) with 3.0 mL/min flow, followed by 20 mL Ultravist 370 with 1.5 mL/min flow. This was followed by irrigation with 50 mL sodium chloride at 3.0 mL/min flow.

### Quantification of skeletal muscle mass

Measurements of SMM were obtained by a trained investigator (T.S., doctoral student) at two time points with an interval of 6 weeks and by an experienced investigator (N.W., in-training, 3 years of experience; H.N., radiologist, 6 years of experience) as a second reader, each independently to determine intra- and inter-rater variability. Measurements were performed on transverse CT scans using IMPAX (IMPAX EE R20; Agfa HealthCare).

**Fig. 1:**
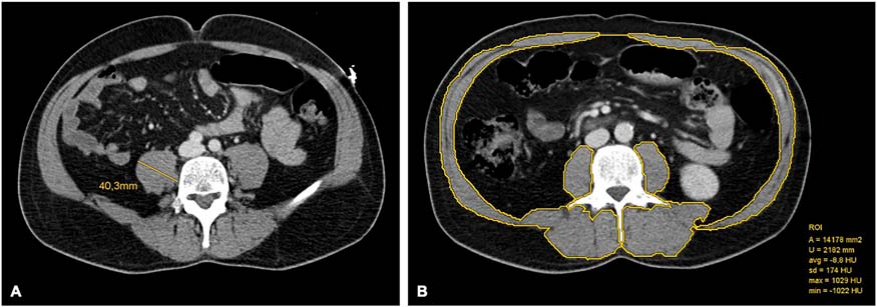
Measurement of skeletal muscle parameters. **A** Measurement of the transverse psoas muscle thickness perpendicular to the largest diameter of the psoas muscle on an axial, contrast-enhanced CT scan at the level of the umbilicus. **B** Measurement of the skeletal muscle area (SMA, cm^2^) on an axial, contrast-enhanced CT scan at the level of the L3 with transverse processes imaged. A, area; avg, average; HU, Hounsfield unit; max, maximum; min, minimum; sd, standard deviation, U, circumference

The PMTH (mm/m) was calculated by measuring the transverse diameter of the right psoas muscle perpendicular to its largest diameter (mm) at the level of the umbilicus and normalizing it to body height (m) (Fig. 1A) [16, 19, 28].

The SMA (cm^2^) was measured manually using the IMPAX tool to segment a contour with computer-assisted edge detection at the level of the L3, with transverse processes imaged (Fig. 1B) and normalized to the square of body height (m^2^) to calculate the SMI (cm^2^/m^2^), as previously described [28]. The psoas, paraspinal, and abdominal wall muscles were included in the selected CT slice [12].

### Statistical analysis

Statistical analyses were performed by F.M. using Statistical Package for the Social Sciences software (SPSS, version 28.0; IBM Corp.). The selection of the sample size was based on previous publications [28]. Continuous variables are presented as mean (± SD) or median with an interquartile range. Continuous data were compared using the Student’s *t*-test when the variables were normally distributed. Continuous nonparametric variables were compared using the Mann–Whitney U test. One-way analysis of variance was used to compare longitudinal variations in continuous variables. Categorical variables were compared using the chi-square or Fisher’s exact test. The Spearman-Rho rank correlation coefficient was calculated to determine the correlation between SMI and PMTH values as well as the inter-rater and intra-rater variability. The significance level was set at *p* < 0.05.

For the final determination of the mean value and SD, patients whose measured values were below the 5th percentile or above the 95th percentile were excluded from the overall cohort. This was performed separately in the SMI and PMTH. In this manner, random outliers were eliminated, and the variance was kept low.

## Results

### Patient characteristics

**Fig. 2:**
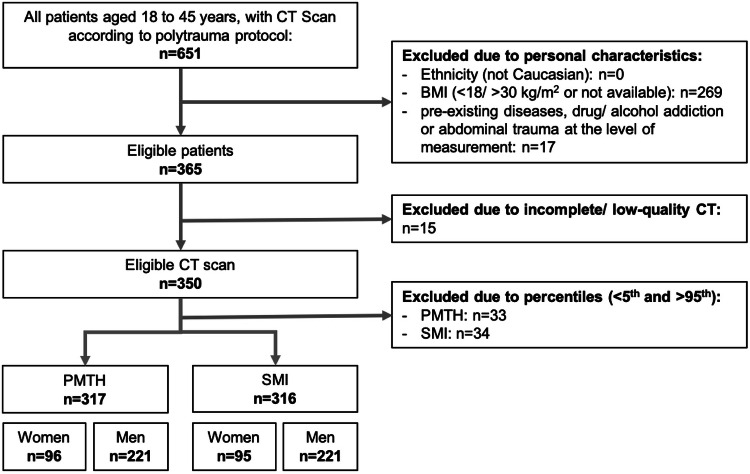
The flowchart shows the initial number of patients and exclusion numbers. n, number

A total of 350 patients who underwent polytrauma CT between 2018 and 2022 met the inclusion criteria (Fig. 2). Participants were predominantly men (70%). The mean age of women and men was comparable, with 31.9 (± 7.9) years and 31.7 (± 7.7) years, respectively. The baseline characteristics, including height, weight, and BMI, were lower in women than in men: height, 1.67 (± 0.07) and 1.80 (± 0.08) m; weight, 64.1 (± 10.7) and 79.4 (± 10.5) kg; and BMI 22.8 (± 2.8) and 24.4 (± 2.5) kg/m^2^, respectively (Table 1). A detailed distribution of patient characteristics according to age groups is presented in Supplementary Table 1. No considerable differences in height, weight, and BMI were found between the individual age groups within the two sexes.

**Table 1:** Participant characteristics depending on sex

	All	Women	Men
*N*	350 (100)	105 (30)	245 (70)
Age (years)	31.7 (± 7.8)	31.9 (± 7.9)	31.7 (± 7.7)
Height (m)	1.76 (± 0.10)	1.67 (± 0.07)	1.80 (± 0.08)
Weight (kg)	74.8 (± 12.7)	64.1 (± 10.7)	79.4 (± 10.5)
BMI (kg/m²)	23.9 (± 2.7)	22.8 (± 2.8)	24.4 (± 2.5)

*n* (%), mean (± SD)

*BMI* body mass index

### Skeletal muscle parameters

**Fig. 3:**
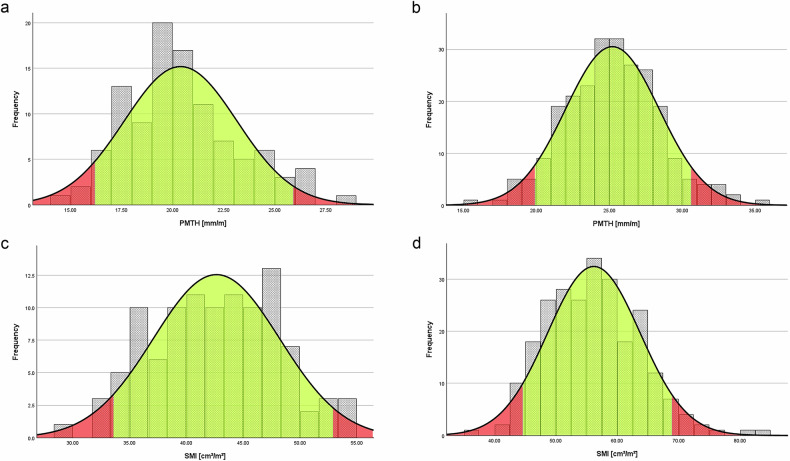
Histograms from (**a**) PMTH (women), (**b**) PMTH (men), (**c**) SMI (women), and (**d**) SMI (men). Red indicates < 5th and > 95th percentiles; green indicates 5th–95th percentiles. PMTH, psoas muscle thickness per height; SMI, skeletal muscle index

Owing to the retrospective nature of the study, the possibility of obtaining patient information was limited. To eliminate outliers due to possibly unknown factors or pre-existing conditions, participants below the 5th percentile or above the 95th percentile were excluded from the calculation of the cutoff value (Fig. 3 and Supplementary Table 2). For example, one woman had an SMI of 29.9 cm^2^/m^2^, which may indicate a non-recognizable pre-existing condition. In contrast, one man had an SMI of 84.4 cm^2^/m^2^, which may suggest excessive bodybuilding.

In the resulting cohorts, the mean values of age, height, weight, and BMI were similar to those in the total cohort of women and men (Supplementary Table 3). In a linear regression analysis, only sex showed a strong positive correlation with muscle mass. The other characteristics did not influence muscle mass in this study’s homogeneous population of young and presumably healthy patients (Supplementary Table 4).

The PMT was 33.8 (± 3.9) mm for the female cohort and 45.5 (± 4.9) mm for the male cohort, and the PMTH was 20.3 (± 2.3) and 25.2 (± 2.5) mm/m, respectively. According to the recommendations of the EWGSOP, two SD below the mean were defined as the cutoff values for sarcopenia. Accordingly, the cutoff values for the PMTH were 15.7 mm/m for women and 20.2 mm/m for men. The SMA was 11,876 (± 1366) and 18,187 (± 2157) mm^2^ for women and men, respectively, and the SMI was 42.6 (± 4.7) and 56.0 (± 6.0) cm^2^/m^2^, respectively. For the SMI, the cutoff value was 33.2 cm^2^/m^2^ for women and 44.0 cm^2^/m^2^ for men (Table 2).

**Table 2:** Muscle mass indices for the 5th–95th percentile depending on sex

	PMTH (women)	PMTH (men)	SMI (women)	SMI (men)
*n*	96 (30.3)	221 (69.7)	95 (30.1)	221 (69.9)
PMT (mm)	33.8 (± 3.9)	45.5 (± 4.9)		
PMT (mm, mean – 2x SD)	26.0	35.7		
PMTH (mm/m)	20.3 (± 2.3)	25.2 (± 2.5)		
**PMTH (mm/m, mean – 2x SD)**	**15.7**	**20.2**		
SMA (mm²)			11,876 (± 1366)	18,187 (± 2157)
SMA (cm^2^, mean – 2x SD)			91.4	138.7
SMI (cm²/m²)			42.6 (± 4.7)	56.0 (± 6.0)
**SMI (cm**^**2**^**/m**^**2**^**, mean – 2x SD)**			**33.2**	**44.0**

Mean – 2x standard deviations is regarded as the threshold between sarcopenia and normal PMTH/SMI

*n* (%); mean (± SD) unless otherwise indicated

*PMT* psoas muscle thickness, *PMTH* psoas muscle thickness per height, *SMA* skeletal muscle area, *SMI* skeletal muscle index

bold = final cutoff

A direct comparison of SMI and PMTH revealed a Spearman-Rho coefficient of 0.694 (*p* < 0.001) and thus a strong to very strong positive correlation (i.e., increasing SMI values correlated with increasing PMTH values).

### Intra- and inter-rater variability

To determine the reliability of the measurement method, measurements were repeated in 100 randomly selected patients (equally distributed by sex) with a 6-week interval from the same reader and from a second reader; no significant differences were found between the different measurements (Table 3). The Spearman-Rho correlation coefficients ranged from 0.780 to 0.981.

**Table 3:** Intra- and inter-rater variability for PMTH and SMI

		1st run	2nd run		2nd rater	
		Mean (± SD)	Mean (± SD)/Spearman-Rho	*p*-value	Mean (± SD)/Spearman-Rho	*p*-value
All	PMTH (mm/m)	22.34 (± 4.09)	22.98 (± 4.14)	0.323*	21.80 (± 4.40)	0.323*
			0.930	< 0.001^#^	0.926	< 0.001^#^
	SMI (cm²/m²)	49.11 (± 9.76)	49.84 (± 9.65)	0.596*	48.42 (± 9.56)	0.554*
			0.981	< 0.001^#^	0.974	< 0.001^#^
Women	PMTH (mm/m)	19.57 (± 2.46)	19.91 (± 2.07)	0.496*	18.82 (± 2.42)	0.155*
			0.780	< 0.001^#^	0.801	< 0.001^#^
	SMI (cm²/m²)	41.83 (± 5.79)	42.79 (± 5.69)	0.392*	41.33 (± 5.58)	0.580*
			0.966	< 0.001^#^	0.960	< 0.001^#^
Men	PMTH (mm/m)	25.11 (± 3.47)	26.06 (± 3.32)	0.212*	24.79 (± 3.89)	0.722*
			0.917	< 0.001^#^	0.850	< 0.001^#^
	SMI (cm²/m²)	56.39 (± 7.11)	56.89 (± 7.38)	0.780*	55.52 (± 7.13)	0.490*
			0.960	< 0.001^#^	0.947	< 0.001^#^

*PMTH* psoas muscle thickness per height, *SMI* skeletal muscle index

* Mann–Whitney U test

^#^ Spearman-Rho correlation

## Discussion and conclusion

To the best of our knowledge, our study is the first to establish cutoff values in a large, homogeneous group of presumably healthy, young patients. We described sex-specific cutoff values for skeletal muscle mass (SMM) measured using CT scans in a presumably healthy Caucasian population of 350 participants. The EWGSOP recommended cutoff values, defined as the mean minus two SD. For the PMTH, these were 15.7 mm/m in women and 20.2 mm/m in men; for the SMI, they were 33.2 cm^2^/m^2^ in women and 44.0 cm^2^/m^2^ in men. Meeting the recommendations of the EWGSOP, we used CT as the gold standard for quantifying SMM. The decisive advantages of this method are the clear separation of muscle and fat and the routine availability of CT images in surgical, oncological, and intensive care patients, which means that there is no additional radiation exposure or financial burden [2].

To date, there is a wide range of published data on cutoff values for SMM, making it difficult to identify patients at risk of sarcopenia. In recent years, the skeletal muscle index (SMI) has proven to be a reliable parameter for determining SMM. However, less data is available for the psoas muscle thickness per height (PMTH), which is much easier and quicker to determine. There are also doubts regarding whether the PMTH reliably reflects the SMM. The PMTH cutoff values in accordance with the European Working Group on Sarcopenia in Older People (EWGSOP) recommendations have not yet been published [2, 29, 30].

However, to determine reduced SMM in these patient groups, cutoff values from a young, healthy population are required. In contrast, it is difficult to obtain CT scans from young, healthy individuals because they usually have no indication for such an examination, and any unnecessary radiation should be avoided. Therefore, we used CT scans from severely injured patients or those with polytrauma, who were otherwise presumably healthy and had no injuries in the measurement field, to determine the cutoff values. The primary methodological strength is the standardized protocol, which ensures consistent application in different clinical settings. It has been previously described that examination parameters may have an impact on measurements. To validate our data, we repeated the measurements after 6 weeks and with a second reader in 100 participants. No significant differences were found. Thus, the intra-rater and inter-rater variabilities were very low, indicating high reliability of the method.

To date, few sex-specific SMI cutoff values for healthy populations have been published. Unfortunately, the methodology for reporting cutoff values differs among studies, making comparisons difficult. The two predominant reporting methods are the mean minus two SD, as suggested by the EWGSOP, and the 5th percentile [15]. In addition, cohorts are defined very differently in terms of demographic and anthropometric data, and often data from organ donors or patients with pre-existing diseases are used to describe a healthy population (Table 4).

**Table 4:** Summary of previous results according to the current literature

Authors	Country/ ethnicity	Population/ patients	Method	*n*	Age (years)	BMI (kg/m^2^)	Results (SMI in cm^2^/m^2^)	Deviation SMI	Limitations
Merboth/ Wotschel	Germany/ Caucasian	Polytrauma patients	Mean − 2 SD, < 5th and > 95th percentile excluded	316 (SMI), 317 (PMTH); 70% male	18–45	18–30	- SMI: 33.2 (f), 44.0 (m) - PMTH (in mm/m): 15.7 (f), 20.2 (m)	N/A	- Unicentric, retrospective design - Predominantly male participants (70%) - Only applicable to the analyzed ethnicity
Van Der Werf et al [12]	Netherlands/Caucasian	Kidney donors	5th percentile	300; 42% male	20–60; subgroup 1: 20–29 and 30–39; subgroup 2: 18–40	17.5–40.7; subgroup 1: 17–20, 20–25 and 25–30; subgroup 2: not indicated	- SMI: 32.7 (f), 43.1 (m); - subgroup 1^a^: 33.5 (f), 42.1 (m); - subgroup 2^b^: 33.0 (f), 44.7 (m)	Subgroup 2: within 0.6% (f), 1.6% (m)	- Retrospective design - Size of subgroups stratified for age and BMI not given, important in view of average age (m = 52 ± 12 years, f = 54 ± 11 years) - Subgroups include morbidly under-/overweight patients - Only applicable to analyzed ethnicity
Derstine et al [13]	USA/no restrictions	Kidney donors	Mean − 2 SD	727; 44% male	18–40	No restrictions	34.4 (f), 45.4 (m)	Within 3.6% (f), 3.2% (m)	- Unicentric, retrospective design - Cutoff values include morbidly under-/overweight patients - Not stratified for ethnicity
Derstine et al [34]	USA/no restrictions	Kidney donors	Mean − 2 SD and 5th percentile	1103; 42% male	18–40	No restrictions	SMI reported normalized to body height (cm^2^/m), and not to square of body height (cm^2^/m^2^)	Not comparable due to different way of reporting SMI	- Unicentric, retrospective design - Reported cutoff values include morbidly under-/overweight patients - Not stratified for ethnicity
Van Vugt et al [35]	Netherlands/Caucasian	Kidney donors	5th percentile	1073; 46% male	Different subgroups; selected subgroups: 20–29 and 30–39	Different subgroups; selected subgroups: 17–20, 20–25 and 25–30	Selected subgroups^a^: 34.3 (f); 42.4 (m)	Within 3.3% (f), 3.6% (m)	- Retrospective design - Size of subgroups stratified for age and BMI not given, important in view of median age (51 years, IQR 41–59 years) - Subgroups include morbidly under-/overweight patients - Only applicable to analyzed ethnicity
Westenberg et al [15]	Netherlands/Caucasian	Kidney donors	Mean − 2 SD	960; 50% male	Different subgroups; selected subgroups: 20–29 and 30–39	Different subgroups; selected subgroup: 25–29.99	Selected subgroups^a^: 43.7 (m), 38.4 (f, 30–39 years, no value given for 20–29 years due to insufficient group size)	Within 0.7% (m), 15.7% (f)	- Unicentric, retrospective design - Size of subgroups stratified for age and BMI not given, important in view of average age (m = 52 ± 12 years, f = 54 ± 10 years) - Subgroups include morbidly under-/overweight patients - Metabolic syndrome in 14.9% of subjects (according to modified criteria of the National Cholesterol Education Program Adult Treatment Panel III, NCEP/ATPIII) - Only applicable to analyzed ethnicity

Despite differences, e.g., in the calculation method, the study population and the CT parameters, most of the SMI cutoff values given are very similar (see column “Deviation SMI”)

*BMI* body mass index, *f* female, *IQR* interquartile range, *m* male, *n* number, *SD* standard deviation

^a^ Self-calculated reference values

^b^ Indicated by authors in the main text

Differences between the published cutoff values can be caused not only by age, sex, BMI, and ethnicity, but also by various other factors. On the participant side, variations in physique, physical activity, lifestyle, culture, and diet have been reported before. On the methodological side, differences in CT parameters, contrast agent use and phases, and experience with muscle area measurements should be considered. Therefore, sex- and ethnicity-specific cutoff values, as well as similar CT protocols and quantification methods, are important for the diagnosis of sarcopenia. Regarding sex-specific differences, our findings are in line with those of previous studies and confirm higher SMI (1.33-fold) and PMTH (1.29-fold) in men than in women [2, 12, 15, 31].

In a recently published meta-analysis, an attempt was made to establish a global cutoff value for the SMI in accordance with the EWGSOP recommendations. 14 studies from 7 different countries were included. However, this revealed an enormous variance and heterogeneity between the individual studies. The lowest SMI cutoff value for men was given as 27.8 cm^2^/m^2^ (India) and the highest as 48.3 cm^2^/m^2^ (USA). The SMI mean value of the 11 non-American studies was not even within the 95% confidence interval of the mean value of the 3 American studies [32]. Our working group is therefore not convinced that it is either possible or sensible to generate a uniform, global cutoff value for muscle mass indices due to the natural differences in the anatomy and physiology of the individual ethnic groups. Considering only the studies with mainly Caucasian patients, the differences are mostly marginal, despite the mentioned limitation of each single study, as shown in Table 4. Most previously published SMI cutoff values for Caucasians differed from our proposed values only up to 3.6%, in other words, ± 1.2 cm^2^/m^2^ for women and ± 1.6 cm^2^/m^2^ for men. The “true” SMI cutoff value for Caucasians is therefore likely to be somewhere in between.

This study had several limitations. It had a unicentric, retrospective design, as in many studies reporting skeletal muscle parameters [12–15, 23, 29, 31, 33]. The analyzed population comprised exclusively of Caucasians. Because SMM differs among ethnicities, our reference values do not apply to other ethnic groups [12, 14]. The tests performed to rule out pre-existing conditions in our study were not as specific as those performed for organ donation. In addition, physical activity, lifestyle, and dietary habits were not recorded in a structured manner. However, the statistical design with the 5th–95th percentile was chosen for this purpose in order to exclude patients with any unknown pre-existing conditions or lifestyle factors that could influence SMM.

Nevertheless, we established sex-specific reference values for psoas muscle thickness per height (PMTH) and skeletal muscle index (SMI) in a Caucasian population according to the European Working Group on Sarcopenia in Older People (EWGSOP) consensus recommendations. We are the first working group that was able to acquire a young and presumably healthy group of patients by following the recommendations with a strict standardized screening protocol and by eliminating random outliers. For the first time, data from CT scans of severely injured patients, instead of organ donors, were used, which may be more representative of a general healthy population. We are also the first to apply the EWGSOP recommendations for determining the reference value for the PMTH. Furthermore, we achieved very low intra- and inter-rater variabilities, demonstrating that PMTH and SMI determination are reliable and accurate methods for measuring skeletal muscle mass. Therefore, it is now time to investigate the cutoff values for the PMTH and SMI in further studies with regard to their significance for clinically relevant outcomes, such as morbidity or mortality in cancer patients.

## Supplementary information

Supplementary information

## Abbreviations

BMI: Body mass index
CT: Computed tomography
EWGSOP: European Working Group on Sarcopenia in Older People
PMTH: Psoas muscle thickness per height
SD: Standard deviation
SMA: Skeletal muscle area
SMI: Skeletal muscle index
SMM: Skeletal muscle mass
