# Supplementary material for: CT-based muscle mass cutoff values for Caucasians according to the European Working Group on Sarcopenia recommendations
Source: Eur Radiol. 2026 Mar 12;36(7):5353–63. doi: 10.1007/s00330-026-12396-9 (PMC13282310; doi:10.1007/s00330-026-12396-9)
Supplement: Supplementary file 1 — Supplementary information [file 330_2026_12396_MOESM1_ESM.pdf]

CT-based muscle mass cut-off values for Caucasians according to the European Working Group on Sarcopenia recommendations

ELECTRONIC SUPPLEMENTARY MATERIAL

	n	All	Women	Men
Height (m)				
18-25 years	85/26/59	1.8 (±0.1)	1.7 (±0.1)	1.8 (±0.1)
25-35 years	128/36/92	1.8 (±0.1)	1.7 (±0.1)	1.8 (±0.1)
35-45 years	137/43/94	1.8 (±0.1)	1.7 (±0.1)	1.8 (±0.1)
Weight (kg)				
18-25 years	85/26/59	71.8 (±12.0)	63.0 (±8.0)	75.7 (±11.4)
25-35 years	128/36/92	75.5 (±12.8)	62.9 (±10.2)	80.4 (±10.1)
35-45 years	137/43/94	76.1 (±12.8)	65.7 (±12.4)	80.8 (±10.0)
BMI (kg/m²)				
18-25 years	85/26/59	23.0 (±2.6)	22.3 (±2.5)	23.3 (±2.6)
25-35 years	128/36/92	24.1 (±2.7)	22.7 (±2.8)	24.6 (±2.4)
35-45 years	137/43/94	24.4 (±2.6)	23.2 (±3.0)	24.9 (±2.2)

Supplementary Table 1. Participant characteristics depending on sex and age distribution. Note: n (%), mean (± SD). BMI, body mass index.

	Women		Men	
PMT (mm)	34.1 (±4.7)		45.5 (±6.1)	
PMTH (mm/m)	20.4 (±2.8)		25.2 (±3.2)	
**PMTH (mm/m, mean - 2 SD)**	**14.8**		**18.8**	
	5th percentile	**16.2397**	5th percentile	**19.9282**
	95th percentile	**25.8637**	95th percentile	**30.6389**
SMA (mm^2)	11900 (±1577)		18243 (±2556)	
SMI (cm^2/m^2)	42.7 (±5.7)		56.2 (±7.5)	
**SMI (cm^2/m^2, mean - 2 SD)**	**31.3**		**41.2**	
	5th percentile	**33.5969**	5th percentile	**44.6156**
	95th percentile	**52.8937**	95th percentile	**68.8707**

Supplementary Table 2. Muscle mass indices for all included patients and the 5th–95th percentile depending on sex. Two standard deviations below the mean were considered the cut-off values between sarcopenia and normal PMTH and SMI.

Note: Mean (± SD), unless otherwise indicated. PMT, psoas muscle thickness; PMTH, psoas muscle thickness per height; SMA, skeletal muscle area; SMI, skeletal muscle index.

	**PMTH (Women)**	**PMTH (Men)**	**SMI (Women)**	**SMI (Men)**
Age (years)	32 (±8)	31.7 (±7.7)	32.3 (±7.7)	31.5 (±7.9)
Height (m)	1.67 (±0.07)	1.81 (±0.08)	1.67 (±0.07)	1.80 (±0.08)
Weight (kg)	63.8 (±10.3)	79.6 (±10.5)	64.0 (±10.9)	79.5 (±10.4)
BMI (kg/m^2)	22.8 (±2.7)	24.4 (±2.5)	22.8 (±2.8)	24.4 (±2.4)

Supplementary Table 3. Additional patient characteristics for the 5th–95th percentile depending on sex and sarcopenia indices.

Note: Mean (± SD), unless otherwise indicated. BMI, body mass index; PMTH, psoas muscle thickness per height; SMI, skeletal muscle index.

	regression coefficient	95% confidence interval		p value
Sex	15.226	13.427	17.025	<0.001
Age	-0.062	-0.146	0.021	0.143
Height	-54.095	-109.389	1.198	0.055
Weight	0.333	-0.333	0.999	0.326
BMI	0.194	-1.886	2.273	0.855

Supplementary Table 4. Linear regression analysis regarding patient characteristics and relation to muscle mass. Only sex showed a strong positive correlation (male sex and higher skeletal muscle index). The remaining characteristics did not influence muscle mass in this study's homogeneous population of young and healthy patients.
Note: BMI, body mass index
